# Malaria Cases in a Tertiary Hospital in Kuala Lumpur, Malaysia: A 16-Year (2005–2020) Retrospective Review

**DOI:** 10.3390/tropicalmed6040177

**Published:** 2021-09-29

**Authors:** Nor Diyana Dian, Ahmad Firdaus Mohd Salleh, Mohd Amirul Fitri A Rahim, Mohd Bakhtiar Munajat, Siti Nor Azreen Abd Manap, Nuraffini Ghazali, Noor Wanie Hassan, Zulkarnain Md Idris

**Affiliations:** 1 Department of Parasitology and Medical Entomology, Faculty of Medicine, Universiti Kebangsaan Malaysia, Kuala Lumpur 56000, Malaysia; P103737@siswa.ukm.edu.my (N.D.D.); P103307@siswa.ukm.edu.my (M.A.F.A.R.); P95556@siswa.ukm.edu.my (M.B.M.); sitinorazreen@ukm.edu.my (S.N.A.A.M.); nuraffini.ghazali@ppukm.ukm.edu.my (N.G.); noor.wanie.hassan@ppukm.ukm.edu.my (N.W.H.); 2 Department of Laboratory Diagnostic Service, Universiti Kebangsaan Malaysia Medical Centre, Kuala Lumpur 56000, Malaysia; ahmad.firdaus.mohd.salleh@ppukm.ukm.edu.my

**Keywords:** malaria, *Plasmodium knowlesi*, trends, retrospective, incidence, Malaysia

## Abstract

While there has been a tremendous decline in malaria disease burden in the remote parts of Malaysia, little is known about malaria incidence in its urban localities. This study aimed to analyze trends of malaria cases in urban Kuala Lumpur, Malaysia. All suspected cases presented to a university hospital in Kuala Lumpur from January 2005 to December 2020 were examined by microscopy. Infection status was analyzed using descriptive statistics and curve estimation analysis. Of 3105 blood films examined, 92 (3%) were microscopically confirmed malaria cases. *Plasmodium vivax* infections accounted for the majority (36.9%) of all malaria cases. Nearly half (47.8%) of cases were found among foreign cases (*p* < 0.001). The majority of foreign cases were male (86.4%) and came from Southeast Asian countries (65.9%). The curve estimation analysis showed significant decreases in malaria cases due to *P. vivax* (R^2^ = 0.598; *p* < 0.001) and *Plasmodium falciparum* (R^2^ = 0.298, *p* = 0.029), but increases for *Plasmodium knowlesi* (R^2^ = 0.325, *p* = 0.021) during the 16 years. This study showed that malaria incidence in urban Kuala Lumpur is low and has remained stable since 2005. However, *P. knowlesi* has played a significant role in the increase in overall malaria in the area, highlighting the importance of continued vigilance and improved surveillance.

## 1. Introduction

Malaysia is a country that is in the pre-elimination phase of malaria and continues to progress towards elimination, having reported only 85 cases of indigenous human malaria cases in 2017 [1]. Even though malaria control activities have significantly reduced human malaria incidence in Malaysia, the resurgence of the monkey malaria parasite *Plasmodium knowlesi* remains a main public health problem in the less-developed areas of the country, especially in Malaysia Borneo [2,3,4] and among hard-to-reach populations of indigenous people (i.e., Orang Asli) in Peninsular Malaysia [5,6,7,8]. About one-third (32%) of total malaria cases occur in Peninsular Malaysia, and the majority of these are found in the central, southeastern and northern coastal regions [9]. The remaining 68 percent of cases are found in Malaysian Borneo in the states of Sabah and Sarawak [10].

Malaysia reoriented its intent from malaria control to elimination in 2011, with a phased goal of achieving zero local transmission in Peninsular Malaysia by 2015, and in Sabah and Sarawak by 2020. Malaysia is vulnerable to malaria importation, primarily from Indonesian and Filipino migrant workers seeking employment in Malaysia’s growing economy [10,11]. In addition, many documented and undocumented migrants from Myanmar, Bangladesh, Nepal, Indonesia and Thailand also enter Peninsular Malaysia to serve the low-skilled and semi-skilled sectors of the economy, especially in the urban areas. In 2014, imported cases accounted for 20 percent of all cases in Malaysia [12]. In countries approaching elimination, imported cases tend to make up most of the recorded cases and threaten the re-establishment of malaria transmission in receptive areas [13]. Thus, the malaria elimination target in Malaysia may be at risk, as the country is largely dependent on foreign workers that come from other Asian countries.

Malaria has been on the notifiable disease list of Malaysia since 1988. It is mandatory for all laboratory-confirmed cases of malaria to be notified to the nearest district health office within 7 days of confirmed diagnosis before sending the data to the central national level [14]. Thus, prompt notification of malaria cases together with malaria control measures have led to a significant decline in malaria disease burden in remote parts of Malaysia. Nonetheless, the trend of malaria incidence based on passive case detection is not well documented in urban localities. Epidemiological data, such as trends of malaria positivity rates at public institutions and hospitals regardless of their locations, are essential to design appropriate interventions. Therefore, this study aims to describe the more recent epidemiological and trend of malaria cases diagnosed in the tertiary care referral and teaching hospital of Kuala Lumpur, Malaysia.

## 2. Materials and Methods

### 2.1. Study Area

This study was conducted at Hospital Canselor Tunku Muhriz (HCTM), a tertiary care referral and teaching hospital of the National University of Malaysia (UKM). As the hospital is a major medical center located in the capital city of Kuala Lumpur, Malaysia, it serves as a proxy measure for the trend of malaria in the urban area, which may contribute to evidence-based decisions on malaria control activities.

### 2.2. Study Design

This retrospective laboratory record review study was carried out to determine 16 years (January 2005–December 2020) of malaria cases. This study was conducted according to the guidelines of the Declaration of Helsinki and approved by the Research and Ethics Committee of UKM (Reference No. UKM PPI/111/8/JEP-2018-055). The need for informed consent was waived by the committee, considering the retrospective nature of the study.

### 2.3. Data Collection

The study participants were all malaria-suspected individuals who had a complaint of febrile illness at HCTM during the study period. Sociodemographic and laboratory data regarding malaria were extracted from the electronic-based reporting system of HCTM and yearly laboratory logbooks from the Department of Parasitology and Medical Entomology, Faculty of Medicine in UKM. Malaria diagnosis was based on microscopic examination of Giemsa-stained thick and thin blood smears, while malaria rapid diagnostic tests and molecular analyses were not performed. In HCTM, microscopic examination is the gold standard diagnostic method for the detection and species identification of *Plasmodium* parasites. Peripheral smear examinations of well-prepared and well-stained thick and thin blood films were used to diagnose malaria in the laboratory. The hospital strictly follows Malaysia’s standard operating procedures in all phases of the quality control for capillary blood sample collection, smear preparation, staining and blood film examination for malaria parasite detection. Blood films were fixed and stained with 3% Giemsa stain and examined under oil emersion (10 × 100 magnification) by trained microscopists. Blood films were defined as negative if no parasites were found after examining 100 high-power microscopy fields. For all positive samples, malaria species were identified, and asexual parasite forms were counted against 500 leukocytes. Parasite density was estimated from parasite counts, assuming that there were 8000 leukocytes per microliter (µL) of blood. For the remaining blood sample of positive cases, multiple blood smears were made and kept for educational purposes and future research.

### 2.4. Statistical Analysis

All the data were merged, cleaned and cross-checked, using a Microsoft Excel version 16.0.4266.1011 spreadsheet. The data were analyzed, using STATA/SE version 13.1 (StataCorp, College Station, TX, USA) and GraphPad Prism version 5.03 (GraphPad Software Inc., San Diego, CA, USA). A descriptive analysis was performed, using Pearson’s Chi-square test and Fisher’s exact test in order to assess the associations between the malaria infection status determined by microscopy and the individual’s general information (i.e., gender, age group, ethnicity and nationality). Meanwhile, the continuous variable (i.e., age) was summarized as median with an interquartile range (IQR) and analyzed, using the Kruskal–Wallis test. Curve estimation linear regression analysis was used to analyze the relationship between malaria positivity rates and year. Yearly proportions of malaria cases were fitted into the curve estimation model, and the coefficient of determination (R^2^) was used to evaluate the correlation between the two variables. A *p* < 0.05 was considered statistically significant.

## 3. Results

Over a period of 16 years (2005–2020), 3105 blood films were requested for malaria diagnosis at HCTM of which 92 (3%; 95% confidence interval [CI]: 2.4–3.6]) were microscopically confirmed malaria cases (Table 1). The median age of the malaria cases was 30 (IQR: 25–39) years. The majority (71.7%, 95% CI: 61.4–80.6) of the infected were young adults (18–40 years) and the age distribution differed significantly between the infected and non-infected groups (*p* = 0.007). Individuals of Bumiputera ethnicity accounted for most of the malaria cases, but there was no significant difference in ethnic distributions between the infected and the non-infected groups. When compared to the non-infected group, malaria infections were significantly more common in males (*p* < 0.001) and among Malaysians (*p* < 0.001).

**Figure 1:**
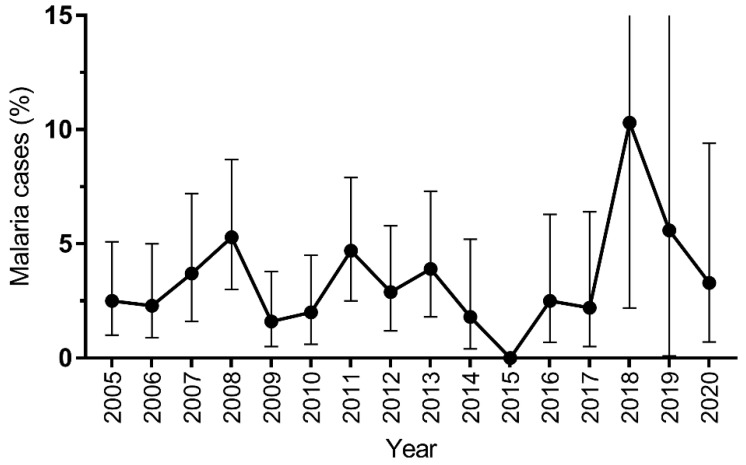
Trend in malaria cases at University Hospital of the National University of Malaysia (UKM) from 2005 to 2020. Error bar represents 95% confidence interval (CI).

The yearly trends of malaria cases are summarized in Figure 1 and Table 2. Malaria cases were reported in all years, except 2015, with the highest prevalence reported in 2018 (10.3%; 3/29) (Figure 1). Despite the apparent fluctuation over the 16 years, no significant difference was observed between the years (*p* = 0.082). Overall, *Plasmodium vivax*, *Plasmodium falciparum*, *P. knowlesi*, *Plasmodium malariae* and mixed *Plasmodium* spp. infections accounted for 36.9% (95% CI: 27.1–47.7), 29.3% (95% CI: 20.3–39.8), 17.4% (95% CI: 10.3–26.7), 4.3% (95% CI: 1.2–10.8) and 11.9% (95% CI: 6.1–20.4) of all malaria cases, respectively. Eleven cases of mixed *Plasmodium* spp. infections were recorded, and no *Plasmodium ovale* infections were observed. *Plasmodium knowlesi* cases were first detected in 2010 and peaked in 2020, accounting for the majority of the cases that year. By nationality, the proportion of malaria cases from local and foreign patients was not significantly different between years (*p* = 0.096), with foreign cases contributed to essentially half (47.8%; 44/92) of all positive cases. Interestingly, out of 44 foreign cases, 38 (86.4%) were males, and 29 (65.9%) came from Southeast Asian countries. There was a total of 3 cases contributed solely by foreign patients in 2014 and a total of 10 cases solely by local patients between 2017 and 2020.

**Figure 2:**
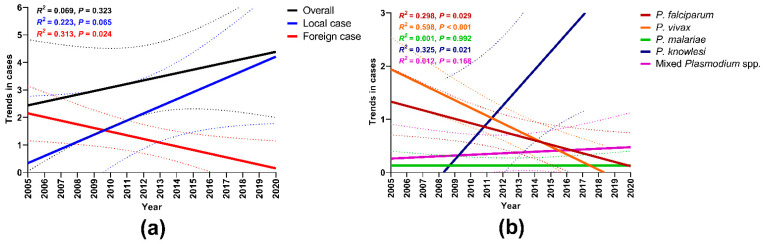
Curve estimation model for malaria cases by (**a**) nationality and (**b**) *Plasmodium* species. Solid lines show model predicted prevalence and broken lines are 95% confidence intervals (CI).

Albeit not significant, the curve estimation analysis using linear models showed a slight increment of overall malaria positivity rates as well as in local cases from 2005 to 2020 (Figure 2a). In contrast, a significant reduction was observed in foreign cases from 2005 to 2020 (R^2^ = 0.313, *p* = 0.024). With regards to *Plasmodium* species (Figure 2b), interestingly, significant reductions were observed for malaria, due to *P. vivax* (R^2^ = 0.598; *p* < 0.001) and *P. falciparum* (R^2^ = 0.298, *p* = 0.029), but not for *P. knowlesi* (R^2^ = 0.325, *p* = 0.021) during the 16 years. As far as these models were used, *P. malariae* and mixed *Plasmodium* spp. infections did not show a significant increase across the years.

## 4. Discussion

Malaysia aims to achieve malaria elimination by the year 2020, and indeed, the drop (98.4%) between 2010 and 2017 from a total of 4731 to 77 recorded indigenous cases for *P. vivax* and *P. falciparum* is highly promising [1]. However, the drop in the number of imported cases over the same period was less encouraging, from a total of 831 to 423 recorded cases [1], and detected zoonotic *P. knowlesi* infections in remote parts of Malaysia have steadily increased [4,15,16,17]. In this study, we examined the available record data from a referral and teaching hospital of UKM located in the capital Kuala Lumpur, Peninsular Malaysia from 2005 to 2020. Over the 16 years, the number of malaria cases diagnosed annually at our hospital has remained relatively low and stable. The present study also revealed that the overall slide positivity rate of malaria was low (i.e., 3%), but higher among males (i.e., 84.8%) and non-Malaysians (i.e., 47.8%). These results are similar to those of studies conducted in the same setting in 2003 [18,19]. With regards to gender, previous studies in Malaysia also showed that males had a higher percentage of malaria cases compared to females, possibly due to occupational exposure, which involves mainly forestry and plantation [4,14]. Malaria species-specific data showed that *P. vivax* was the most prevalent species particularly in foreign cases, with the estimated incidence showed a significant reduction over time [1]. Moreover, the emergence of *P. knowlesi* infections in 2010 among local cases signifies the alarming threat of zoonotic malaria in the country and may hinder malaria elimination efforts.

As one of the fastest-growing cities in Asia, where thousands of foreign workers arrive every year, Kuala Lumpur represents a likely hotspot for malaria importation in Malaysia. Our hospital in Kuala Lumpur received 44 (47.8%) confirmed malaria cases from foreigners over the period of 16 years (January 2005 to December 2020) of which 86.4% were males and 65.9% were from neighboring Southeast Asia countries. This finding is in line with the studies conducted in other Asian countries that highly rely on foreign workers, such as Singapore [20,21], South Korea [22], Japan [23], Kuwait [24], Saudi Arabia [25], Qatar [26], and the United Arab Emirates [27]. In 2017, imported cases in Malaysia accounted for 10.3% of all cases in the country [1]. In 2020, more than 1.8 million migrants/foreign workers registered in Malaysia. These migrants/foreign workers come from 12 different Asian countries, with approximately 1.4 million of them being males (Department of Labor Peninsular, Ministry of Human Resources Malaysia, 2020). Rapid development in the city has led to an influx of low- and semi-skilled foreign workers, many of whom have come illegally or without work permits. In addition, there are significant numbers of displaced people in Kuala Lumpur with no nationality that arrive from malaria-endemic countries in Asia, particularly from Myanmar. Our finding also revealed that 16 out of 29 confirmed malaria cases from Southeast Asia were from Myanmar’s patients. As of January 2021, of the approximately 164,620 refugees and asylum-seekers registered with the United Nations High Commissioner for Refugees (UNHCR) in Malaysia, 86.5% were from Myanmar, 67% were males, and 16.8% of them had resettled in Kuala Lumpur [19]. As Malaysia moves toward elimination, malaria will begin to cluster among certain high-risk groups, including migrants and displaced populations. Improved surveillance, collaboration with key industries and other government agencies, and cross-border cooperation with neighboring endemic countries are critical for addressing the ongoing threat of malaria importation and achieving elimination.

*Plasmodium vivax* was the most common *Plasmodium* species observed in this study. This finding is similar to what was reported in other retrospective studies conducted in Peninsular Malaysia [7,18,28,29,30,31]. For the past 10 years, *P. vivax* has been the main cause of human malaria in Malaysia, and it continues to be a public health concern [1,29]. In 2010, of the 5819 reported cases, 58.2% were due to *P. vivax* [1]. The ability of *P. vivax* to remain dormant in the liver as hypnozoites that can cause relapse following a primary infection, greater asymptomatic asexual carriage, and early gametocyte production provide far greater challenges for malaria elimination in the country. Nevertheless, no case of *P. vivax* was recorded in our hospital from 2015 to 2020 and based on the curve estimation analysis model, there was a significant reduction in *P. vivax* cases over the 16 years. This declining trend is a testament to the commitment of the government and other parties in Malaysia. The Malaysian government launched the National Malaria Elimination Strategic Plan 2011–2020 with the final goal of the complete elimination of locally-acquired malaria (excluding *P. knowlesi*) in Peninsular Malaysia by 2015 and in East Malaysia by 2020 [10]. The national strategic malaria elimination plan currently outlines seven key actions to achieve the elimination goal, including strengthening malaria surveillance system through an online system, intensifying control activities by indoor residual spray (IRS) and insecticide-treated nets (ITN), ensuring early case investigation, prompt treatment and outbreak management as well as improving community awareness and knowledge of malaria [32]. These concerted efforts have resulted in a significant reduction in overall malaria incidence, particularly *P. vivax* cases, over the last decade in Malaysia.

Our work has provided insight into *P. knowlesi* cases in an urban area. Although the greatest number of *P. knowlesi* cases has been reported in remote areas in East Malaysia [2,9,12,33], the infection is also the predominant cause of malaria in Peninsular Malaysia [6,8]. It is unlikely that patients admitted to our hospital acquired the *P. knowlesi* infection in the capital Kuala Lumpur (Federal Territory), which is considered a malaria-free area. However, it is interesting to note that Kuala Lumpur is located within the State of Selangor, a malaria-endemic area in Peninsular Malaysia. In Selangor, local malaria transmission is still being reported from a few districts that adjoin sub-urban and forest range areas with rapid development and deforestation [31,34]. A similar link between deforestation and *P. knowlesi* malaria transmission was observed recently in the State of Sabah, Malaysia Borneo [35,36,37]. It has previously been described that land-use and land-cover changes, including deforestation and urbanization, affect the vector ecologies as a result of the increased sunlight on *Anopheles* breeding sites, species distribution and behavioral capacities [38,39]. With regard to individual-level risk factors, except for a comprehensive case-control study by Grigg et al. on human-related factors in acquiring *P. knowlesi* among rural communities in East Malaysia [40], no study has been conducted in Peninsular Malaysia. Furthermore, human behavioral factors may also be associated with acquiring *P. knowlesi* malaria. Activities appealing to urban populations, such as jungle tracking and camping as well as waterfall picnicking and fishing, may increase exposure to environmental factors conducive to zoonotic transmission of *P. knowlesi* malaria. More detailed evidence about the risk of transmission in urban settings is required to design appropriate interventions. In addition, there is also a need to improve current public health policies to better understand the cause and consequence of changing epidemiological patterns of zoonotic malaria in urban/peri-urban contexts in the country.

## 5. Conclusions

Collectively, the malaria positivity rate in HCTM is low and declining. The declining trend of the overall rate could be due to the significant decline in human malaria cases, particularly due to *P. vivax* and *P. falciparum* infections. However, malaria cases remain a public health concern in the urban setting with the influx of migrant workers and the increasing number of cases with *P. knowlesi* infections. On the other hand, because this study included only a single institution of malaria record review, the data cannot be used to generalize the overall malaria cases for different demographic groups in Kuala Lumpur. Further investigation particularly on more recent data from other hospitals in the city is required in order to assess whether specific groups are underrepresented by the hospital record system. Despite this limitation, this study illustrates that malaria case notification and interventions in Malaysia should be strengthened and highlights the need for further reinforcement to achieve elimination.

## Figures and Tables

**Table 1:** Demographic characteristic of patients screened at University Hospital of the National University of Malaysia (UKM) over 16 years (2005–2020).

Characteristics	Without Malaria	With Malaria	*p*-Value ^a^
No. of patients, N	3013	92	
Median age ^b^ (IQR), years	32 (24–48)	30 (25–39)	0.159
Age group, n (%)			
≤6	67 (2.5)	4 (4.4)	0.007
7–17	200 (7.4)	3 (3.3)	
18–40	1501 (55.8)	66 (71.7)	
>40	921 (34.3)	19 (20.6)	
Gender ^b^, n (%)			
Male	1817 (61.9)	78 (84.8)	<0.001
Female	1116 (38.1)	14 (15.2)	
Malaysia’s ethnic group, n (%)			
Bumiputera	1592 (68.9)	30 (62.5)	0.361
Chinese	524 (22.7)	15 (31.3)	
Indian	193 (8.4)	3 (6.2)	
Nationality ^b^, n (%)			
Malaysian	2309 (81.2)	48 (52.2)	<0.001
Non-Malaysian	534 (18.8)	44 (47.8)	

IQR, interquartile range. ^a^ Chi-square test, Fisher’s exact test or Kruskal–Wallis test comparing with and without malaria. ^b^ No data recorded for age (n = 324), gender (n = 80) and nationality (n = 170).

**Table 2:** Number of malaria cases per year reported at University Hospital of the National University of Malaysia (UKM) in 2005–2020.

Characteristics	Overall	2005	2006	2007	2008	2009	2010	2011	2012	2013	2014	2015	2016	2017	2018	2019	2020
Total number tested, N	3105	276	256	214	281	305	255	277	245	230	166	168	160	135	29	18	90
No. of positive malaria cases, n	92	7	6	8	15	5	5	13	7	9	3	0	4	3	3	1	3
No. of cases by species, n (%)																	
*Plasmodium falciparum*	27 (0.9)	2 (0.7)	4 (1.6)	3 (1.4)	4 (1.4)	1 (0.3)	0 (0.0)	5 (1.8)	1 (0.8)	4 (1.7)	1 (0.6)	0 (0.0)	2 (1.3)	0 (0.0)	0 (0.0)	0 (0.0)	0 (0.0)
*Plasmodium vivax*	34 (1.1)	5 (1.8)	2 (0.8)	5 (2.3)	8 (2.8)	3 (0.9)	2 (0.8)	3 (1.1)	1 (0.8)	4 (1.7)	1 (0.6)	0 (0.0)	0 (0.0)	0 (0.0)	0 (0.0)	0 (0.0)	0 (0.0)
*Plasmodium malariae*	4 (0.1)	0 (0.0)	0 (0.0)	0 (0.0)	0 (0.0)	1 (0.3)	0 (0.0)	1 (0.4)	1 (0.8)	0 (0.0)	0 (0.0)	0 (0.0)	1 (0.6)	0 (0.0)	0 (0.0)	0 (0.0)	0 (0.0)
*Plasmodium knowlesi*	16 (0.5)	0 (0.0)	0 (0.0)	0 (0.0)	0 (0.0)	0 (0.0)	1 (0.4)	4 (1.4)	3 (1.2)	0 (0.0)	1 (0.6)	0 (0.0)	0 (0.0)	0 (0.0)	3 (10.3)	1 (5.6)	3 (3.3)
*Plasmodium ovale*	0 (0.0)	0 (0.0)	0 (0.0)	0 (0.0)	0 (0.0)	0 (0.0)	0 (0.0)	0 (0.0)	0 (0.0)	0 (0.0)	0 (0.0)	0 (0.0)	0 (0.0)	0 (0.0)	0 (0.0)	0 (0.0)	0 (0.0)
Mixed *Plasmodium* spp. ^a^	11 (0.4)	0 (0.0)	0 (0.0)	0 (0.0)	3 (1.1)	0 (0.0)	2 (0.8)	0 (0.0)	1 (0.8)	1 (0.4)	0 (0.0)	0 (0.0)	1 (0.6)	3 (2.2)	0 (0.0)	0 (0.0)	0 (0.0)
No. of cases by age group, n (%)																	
≤6	4 (0.1)	0 (0.0)	0 (0.0)	0 (0.0)	2 (0.7)	0 (0.0)	2 (0.8)	0 (0.0)	0 (0.0)	0 (0.0)	0 (0.0)	0 (0.0)	0 (0.0)	0 (0.0)	0 (0.0)	0 (0.0)	0 (0.0)
7–17	3 (0.1)	0 (0.0)	0 (0.0)	0 (0.0)	0 (0.0)	0 (0.0)	1 (0.4)	1 (0.4)	0 (0.0)	1 (0.4)	0 (0.0)	0 (0.0)	0 (0.0)	0 (0.0)	0 (0.0)	0 (0.0)	0 (0.0)
18–40	66 (2.1)	7 (2.5)	5 (1.9)	8 (3.7)	10 (3.6)	3 (0.9)	2 (0.8)	8 (2.9)	5 (2.1)	8 (3.5)	3 (1.8)	0 (0.0)	3 (1.9)	1 (0.7)	1 (3.5)	0 (0.0)	2 (2.2)
>40	19 (0.6)	0 (0.0)	1 (0.4)	0 (0.0)	3 (1.1)	2 (0.7)	0 (0.0)	4 (1.4)	2 (0.8)	0 (0.0)	0 (0.0)	0 (0.0)	1 (0.6)	2 (1.5)	2 (6.8)	1 (5.6)	1 (1.1)
No. of cases by gender, n (%)																	
Male ^b^	78 (2.5)	6 (2.1)	4 (1.6)	7 (3.2)	12 (4.2)	4 (1.3)	5 (1.9)	12 (4.3)	7 (2.9)	8 (3.5)	2 (1.2)	0 (0.0)	3 (1.9)	2 (1.5)	3 (10.3)	1 (5.6)	2 (2.2)
Female	14 (0.5)	1 (0.4)	2 (0.8)	1 (0.5)	3 (1.1)	1 (0.3)	0 (0.0)	1 (0.4)	0 (0.0)	1 (0.4)	1 (0.6)	0 (0.0)	1 (0.6)	1 (0.7)	0 (0.0)	0 (0.0)	1 (1.1)
No. of cases by Malaysia’s ethnic group, n (%)																	
Bumiputera	30 (0.9)	2 (0.7)	3 (1.3)	1 (0.5)	7 (2.5)	1 (0.3)	1 (0.4)	3 (1.1)	2 (0.8)	0 (0.0)	0 (0.0)	0 (0.0)	3 (1.9)	2 (1.5)	3 (10.3)	0 (0.0)	2 (2.2)
Chinese	15 (0.5)	1 (0.4)	0 (0.0)	0 (0.0)	2 (0.7)	2 (0.7)	0 (0.0)	4 (1.4)	2 (0.8)	1 (0.4)	0 (0.0)	0 (0.0)	0 (0.0)	1 (0.7)	0 (0.0)	1 (5.6)	1 (1.1)
Indian	3 (0.1)	1 (0.4)	0 (0.0)	0 (0.0)	0 (0.0)	1 (0.3)	0 (0.0)	1 (0.4)	0 (0.0)	0 (0.0)	0 (0.0)	0 (0.0)	0 (0.0)	0 (0.0)	0 (0.0)	0 (0.0)	0 (0.0)
No. of cases by nationality, n (%)																	
Malaysian ^c^	48 (1.5)	4 (1.4)	3 (1.3)	1 (0.5)	9 (3.2)	4 (1.3)	1 (0.4)	8 (2.9)	4 (1.7)	1 (0.4)	0 (0.0)	0 (0.0)	3 (1.9)	3 (2.2)	3 (10.3)	1 (5.6)	3 (3.3)
Non-Malaysian ^d^	44 (1.4)	3 (1.1)	3 (1.3)	7 (3.2)	6 (2.1)	1 (0.3)	4 (1.5)	5 (1.8)	3 (1.2)	8 (3.5)	3 (1.8)	0 (0.0)	1 (0.6)	0 (0.0)	0 (0.0)	0 (0.0)	0 (0.0)
No. of cases among non-Malaysian, n (%)																	
Asian ^e^	37 (1.2)	3 (1.1)	2 (0.8)	6 (2.8)	5 (1.8)	1 (0.3)	4 (1.5)	4 (1.4)	3 (1.2)	5 (2.2)	3 (1.8)	0 (0.0)	0 (0.0)	0 (0.0)	0 (0.0)	0 (0.0)	0 (0.0)
African ^f^	7 (0.3)	0 (0.0)	0 (0.0)	1 (0.5)	1 (0.4)	0 (0.0)	0 (0.0)	1 (0.4)	0 (0.0)	3 (0.9)	0 (0.0)	0 (0.0)	1 (0.6)	0 (0.0)	0 (0.0)	0 (0.0)	0 (0.0)

^a^ Total cases of mixed Plasmodium spp. infections: *P. falciparum*/*P. vivax* (n = 3), *P. falciparum*/*P. malariae* (n = 3); *P. vivax*/P.malariae (n = 1), *P. falciparum*/*P. knowlesi* (n = 1), *P. malariae*/*P. knowlesi* (n = 2) and *P. falciparum*/*P. malariae*/*P. knowlesi* (n = 1). ^b^ Total male cases aged 18 and above: Malaysian (n = 38) and non-Malaysian (n = 34). Of the non-Malaysian males, 20 (59%) from Southeast Asia countries, namely from Indonesia (n = 10), Myanmar (n = 9) and Vietnam (n = 1). ^c^ Total malaria by species among Malaysian: *P. falciparum* (n = 11), *P. vivax* (n = 14), *P. malariae* (n = 2), *P. knowlesi* (n = 14) and mixed species (n = 7). ^d^ Total malaria by species among non-Malaysian: *P. falciparum* (n = 16), *P. vivax* (n = 20), *P. malariae* (n = 2), *P. knowlesi* (n = 2) and mixed species (n = 4). ^e^ Total cases by Asian countries: Myanmar (n = 16), Indonesia (n = 11), Pakistan (n = 3); Nepal (n = 2) and others (n = 5). ^f^ Total cases by African countries: Nigeria (n = 3), Sudan (n = 3) and Ghana (n = 1).

## Data Availability

Data are available upon request.
